# Simulation exercises and after action reviews – analysis of outputs during 2016–2019 to strengthen global health emergency preparedness and response

**DOI:** 10.1186/s12992-020-00632-w

**Published:** 2020-12-01

**Authors:** Frederik Anton Copper, Landry Ndriko Mayigane, Yingxin Pei, Denis Charles, Thanh Nam Nguyen, Candice Vente, Cindy Chiu de Vázquez, Allan Bell, Hilary Kagume Njenge, Nirmal Kandel, Zheng Jie Marc Ho, Abbas Omaar, Stéphane de la Rocque, Stella Chungong

**Affiliations:** 1 Country Simulation Exercises & Reviews (CER), World Health Organization Headquarters, 20 Avenue Appia, CH-1211 Geneva, Switzerland; 2 Health Security Preparedness (HSP), World Health Organization Headquarters, 20 Avenue Appia, CH-1211 Geneva, Switzerland

**Keywords:** International health regulations, International health regulations monitoring and evaluation framework, After action review, Simulation exercises, Intra action review, Global health security, Public health emergency preparedness and response, Public health emergency of international concern

## Abstract

**Background:**

Under the International Health Regulations (2005) [IHR (2005)] Monitoring and Evaluation Framework, after action reviews (AAR) and simulation exercises (SimEx) are two critical components which measure the functionality of a country’s health emergency preparedness and response under a “real-life” event or simulated situation. The objective of this study was to describe the AAR and SimEx supported by the World Health Organization (WHO) globally in 2016–2019.

**Methods:**

In 2016–2019, WHO supported 63 AAR and 117 SimEx, of which 42 (66.7%) AAR reports and 56 (47.9%) SimEx reports were available. We extracted key information from these reports and created two central databases for AAR and SimEx, respectively. We conducted descriptive analysis and linked the findings according to the 13 IHR (2005) core capacities.

**Results:**

Among the 42 AAR and 56 SimEx available reports, AAR and SimEx were most commonly conducted in the WHO African Region (AAR: *n* = 32, 76.2%; SimEx: n = 32, 52.5%). The most common public health events reviewed or tested in AAR and SimEx, respectively, were epidemics and pandemics (AAR: *n* = 38, 90.5%; SimEx: *n* = 46, 82.1%). For AAR, 10 (76.9%) of the 13 IHR core capacities were reviewed at least once, with no AAR conducted for food safety, chemical events, and radiation emergencies, among the reports available. For SimEx, all 13 (100.0%) IHR capacities were tested at least once. For AAR, the most commonly reviewed IHR core capacities were health services provision (*n* = 41, 97.6%), risk communication (*n* = 39, 92.9%), national health emergency framework (*n* = 39, 92.9%), surveillance (*n* = 37, 88.1%) and laboratory (*n* = 35, 83.3%). For SimEx, the most commonly tested IHR core capacity were national health emergency framework (*n* = 56, 91.1%), followed by risk communication (*n* = 48, 85.7%), IHR coordination and national IHR focal point functions (*n* = 45, 80.4%), surveillance (*n* = 31, 55.4%), and health service provision (*n* = 29, 51.8%). For AAR, the median timeframe between the end of the event and AAR was 125 days (range = 25–399 days).

**Conclusions:**

WHO has recently published guidance for the planning, execution, and follow-up of AAR and SimEx. Through the guidance and the simplified reporting format provided, we hope to see more countries conduct AAR and SimEx and standardization in their methodology, practice, reporting and follow-up.

**Supplementary information:**

**Supplementary information** accompanies this paper at 10.1186/s12992-020-00632-w.

## Background

Under the International Health Regulations (2005) [IHR (2005)], 194 Members States of the World Health Organization (WHO) and two other State Parties (Liechtenstein and the Holy See) are legally required to develop and maintain minimum core capacities to detect, assess, notify, and respond to any potential public health emergency of international concern (PHEIC) [1, 2]. Public health emergencies may be caused by emerging and re-emerging infectious disease outbreaks, natural disasters, social unrest and conflict, food contamination, or industrial accident including chemical or radioactive nuclear spills, among other hazard risks.

Following the Ebola virus disease (EVD) outbreak in West Africa in 2014–16, at the 68th World Health Assembly (WHA) in 2015, the IHR Review Committee recommended “*to move from exclusive self-evaluation, to approaches that combine self-evaluation, peer review and voluntary external evaluations involving a combination of domestic and independent experts*” [3]. In addition, the IHR Review Committee also recommended for States Parties to urgently implement in-depth reviews of significant disease outbreaks and public health events (PHEs). Consequently, the WHO secretariat developed the IHR Monitoring and Evaluation Framework (IHR MEF) [4], which consists of four complementary components: one mandatory – the States Parties Annual Report (SPAR); and three voluntary – Joint External Evaluations (JEE), After Action Reviews (AAR) and Simulation Exercises (SimEx).

Among the four components of the IHR MEF, AAR and SimEx are the two components that can assess the functionality of a country’s health emergency preparedness and response under an actual “real-life” event or a simulated situation [4]. Both components are widely recognized and used by Member States, WHO and partner organizations as key system improvement and learning tools in emergency management. An AAR provides a means to observe and review actions undertaken in response to a real event of public health concern. It focuses on bringing together key stakeholders involved in the response for collective learning, identifying and documenting lessons learned and challenges, and institutionalizing best practices seen during the response [5–7]. A SimEx is a form of practice, training, monitoring or evaluation of capabilities, involving the description or simulation of an emergency to which a described or simulated response is made [7, 8]. Both AAR and SimEx have well established and internationally recognized standard methodologies, including from WHO [5–8], as well as from partner organizations [9–18]. They aim to test system functionality and coordination, with the results being a set of recommendations of activities proposed and prioritized by the country itself, thus ultimately promotes ownership and enhance public health preparedness and response.

AAR provides an unparalleled opportunity for reflection and collective learning after a real infectious disease outbreak or other public health emergencies [5]. Emerging and re-emerging infectious diseases pose a continuous threat to humanity, and have become increasingly frequent as the human-animal interface becomes more interlaced [19, 20]. It is in the interest of all countries to invest in global health security, given these threats can have significant social and economic ramifications. The outbreak of EVD that occurred in West Africa in 2014–2016 not only caused 11,308 deaths in Guinea, Liberia and Sierra Leone, as of 27 March 2016 [21], but was also estimated to have resulted in a cost of $53.19 billion (2014 USD) [22]. Currently, the world is also facing an unprecedented Coronavirus disease 2019 (COVID-19) pandemic that was declared a PHEIC by WHO on 30 January 2020 [23]. The COVID-19 pandemic has caused global social and economic disruptions with more than 38 million cases and one million deaths as of 14 October 2020 [24], with international travel and trade having been disrupted significantly.

Since the start of the COVID-19 pandemic, multiple countries have used and benefitted from SimEx to enhance their COVID-19 preparedness and response. Many more countries have also been implementing SimEx as part of regular monitoring and evaluation of national IHR (2005) to enhance health security preparedness. These include table top exercises and full-scale exercises, which tested the national capacity to managing potential importation of COVID-19 or other emerging infectious disease outbreak. The lessons and recommendations that emerged from the exercise have supported various aspects of the COVID-19 preparedness and response, including strengthening national testing capacity, early detection of COVID-19 cases, and enhancing preparedness at Points of Entries (PoEs) and among frontline workers [25].

With the current COVID-19 pandemic, it is critical for countries to continually reflect on their ongoing response strategies and adapt their approach as needed to strengthen preparedness and response capacities. WHO through the recently published guidance and tools for the Country COVID-19 Intra-Action Reviews (IARs) advises countries to conduct regular reviews of COVID-19 preparedness and response strategies both at the national and subnational levels to support countries to better control the COVID-19 outbreak, protect the most vulnerable groups in the society and mitigate the impact of the pandemic on livelihoods and economies. COVID-19 IARs provide critical opportunities for learning and implementing practical steps for immediate remediation and improvement of the ongoing response [26].

The importance of COVID-19 IARs was further highlighted during the fourth meeting of the International Health Regulations (2005) Emergency Committee regarding the outbreak of novel coronavirus (2019-nCoV) convened by the WHO Director-General on 31 July 2020, and that issued temporary recommendations to encourage countries to “share best practices, including from intra-action reviews, with WHO; apply lessons learned from countries that are successfully re-opening their societies (including businesses, schools, and other services) and mitigating resurgence of COVID-19” [27]. This necessity to conduct COVID-19 IARs during the ongoing pandemic was also echoed by WHO at all levels in a commentary in Lancet Global Health on 8 Oct 2020 [28].

SimEx are important activities for countries to implement given there are many scenarios that may be rare but important to prepare for. These can include bioterrorism threats, chemical and radiological accidents, as well as a pandemic of a novel strain of virus such as the current COVID-19 outbreak [29–32]. Because emergencies can strike anywhere and at anytime, and disease outbreaks do not respect national boundaries in our inter-dependent and inter-connected world, the ability to respond even in the most remote areas of the world is essential for effective emergency response. Preparing for and responding effectively to such emergencies are among the most pressing challenges facing the international community.

WHO and partner organizations play an important role in both technical and financial support to countries in the planning and implementation of all four components of the IHR MEF and in developing their IHR core capacities, including through SimEx and AAR. Analysis of this information can promote a more evidence-based approach to assess and monitor effective IHR core capacities in “real-life” or simulated situations, and thereby strengthen national and global preparedness. The objectives of this study are to compile all the available AAR and SimEx conducted from 2016 to 2019 into a database, and to summarize this information descriptively and in accordance to the 13 IHR core capacities (Table 1).

**Table 1:** Core capacities as outlined in the International Health Regulations (2005)

C**apacity** N**o**.	IHR C**ore** C**apacities**
C1	Legislation and Financing
C2	IHR Coordination and National IHR Focal Point Functions
C3	Zoonotic Events and the Human-Animal Interface
C4	Food Safety
C5	Laboratory
C6	Surveillance
C7	Human Resources
C8	National Health Emergency Framework
C9	Health Service Provision
C10	Risk Communication
C11	Points of Entry (PoE)
C12	Chemical Events
C13	Radiation Emergencies

*IHR* International Health Regulations, *PoE* Points of entry

## Methods

### Data sources and database compilation

Among the 63 AAR and 117 SimEx supported by WHO from February 2016 to December 2019, 42 AAR and 56 SimEx reports were reported and submitted to the WHO and available to the authors. We extracted key information from these reports into two central databases and assigned each AAR and SimEx with unique identifiers. For the AAR database, we extracted 24 variables from the AAR reports. For the SimEx database, we extracted 20 variables from the SimEx reports. Variables extracted included geographical location based on WHO-designated regions, including [African Region (AFR), American Region (AMR), Eastern Mediterranean Region (EMR), European Region (EUR), South-East Asia Region (SEAR), and Western Pacific Region (WPR)]; date conducted; types of AAR (debrief format, key Informant Interview format, working group format, and mix method format); types of SimEx (tabletop exercise, drill, functional or full scale/field exercise); type of PHE reviewed/tested in AAR and SimEx, respectively; and the date of the end of the event or response (for AAR only) (Please see Supplementary Tables 1 and 2 for the full list of variables extracted from AAR and SimEx reports, respectively).

### Data coding and analysis

For the AAR and SimEx databases, we recoded existing variables into 26 and 27 new variables, respectively. These new variables included the 13 IHR core capacities reviewed or tested (Table 1), and the kind of PHE reviewed or tested in AAR and SimEx, which was coded into three categories: 1) epidemics and pandemics, 2) human-induced/societal and 3) natural disaster. Given AAR and SimEx reports often did not explicitly mention the IHR core capacities reviewed/tested, two independent coders reviewed the reports and coded the IHR core capacities reviewed/tested in each report and obtained consensus by discussing with a team of WHO experts in public health emergency preparedness and response. For AAR reports where the date of the end of the PHE or response were available, we also calculated the timeframe it took to conduct an AAR.

We conducted a descriptive analysis and described the AAR and SimEx conducted from 2016 to 2019 by WHO region, year conducted, AAR format or SimEx type used, and the PHE reviewed or tested, respectively. We also examined the IHR core capacities reviewed or tested in the AAR and SimEx conducted to enable the analysis of global trends, as well as cross-referencing and complementing the findings with other IHR MEF components. Compatibility between gaps identified and formulated recommendations for different IHR core capacities were paired and compared qualitatively.

For categorical data, we presented results either as frequency and percentages, for normally distributed continuous data, we presented these as mean and standard deviation (SD). For continuous data which is not normally distributed, we presented these as median and range.

## Results

### Characteristics of AAR and SimEx

Among the 63 AAR and 117 SimEx supported by WHO from February 2016 to December 2019, WHO received 42 (66.7%) AAR and 56 (47.9%) SimEx reports from the Member States (Table 2). Among the 42 AAR where reports were available, AAR were most commonly conducted in the AFR (*n* = 32, 76.2%), followed by the EUR (*n* = 5, 11.9%), the EMR (n = 3, 7.1%) and the WPR (*n* = 2, 4.8%; Table 2). No AAR reports were received from the AMR and SEAR. Of the 42 AAR reports received, 12 (28.6%) were received for AAR conducted in 2017, 19 (45.2%) were received for AAR conducted in 2018, and 11 (26.2%) were received for AAR conducted in 2019 (Table 2). No AAR reports were available for 2016. Among these 42 AAR, the most common type of AAR reported was the working group format (*n* = 34, 81.0%), followed by the debrief format (*n* = 4, 9.5%), and the mix-method format (*n* = 1, 2.4%; Table 2). Among the 42 AAR reports received, none used the key informant interview format alone, and 3 (7.4%) AAR reports did not describe the formats used.

**Table 2:** Characteristics of the WHO-supported AAR and SimEx conducted worldwide where reports were available, February 2016 to December 2019

AAR (***n*** = 42)			S**im**E**x** (***n*** = 56^**a**^)		
	No.	%		No.	%
**WHO Region**^**b**^			**WHO Region**^**a, b, c**^		
AFR	32	(76.2%)	AFR	32	(52.5%)
AMR	–	–	AMR	1	(1.6%)
EMR	3	(7.1%)	EMR	8	(13.1%)
EUR	5	(11.9%)	EUR	12	(19.7%)
SEAR	–	–	SEAR	2	(3.3%)
WPR	2	(4.8%)	WPR	6	(9.8%)
**Year AAR conducted**			**Year SimEx conducted**		
2016	–	–	2016	16	(28.6%)
2017	12	(28.6%)	2017	11	(19.6%)
2018	19	(45.2%)	2018	17	(30.4%)
2019	11	(26.2%)	2019	12	(21.4%)
**Type of AAR format**			**Type of SimEx used**^**d**^		
Debrief AAR	4	(9.5%)	Table top exercise	36	(62.1%)
Working group AAR	34	(81.0%)	Drill	2	(3.4%)
Key informant interview AAR	–	–	Functional exercise	10	(17.2%)
Mixed-method AAR	1	(2.4%)	Field/full-scale exercise	10	(17.2%)
Unknown^e^	3	(7.1%)	Unknown	–	–
**Public health event category reviewed**^**f**^			**Public health event category tested**		
Epidemics and pandemics	38	(90.5%)	Epidemics and pandemics	46	(82.1%)
Natural disasters	3	(7.1%)	Natural disasters	6	(10.7%)
Human-induced/ Societal	1	(2.4%)	Human-induced/ Societal	4	(7.1%)

*AAR* after action review*, SimEx* simulation exercise, *WHO* World Health Organization, *AFR* African region, *AMR* Region of the Americas, *SEAR* South-East Asia Region, *EUR* European Region, *EMR* Eastern Mediterranean Region, *WPR* Western Pacific Region

^a^ One SimEx report was a global functional exercise with 33 countries from the 6 WHO regions

^b^ Member States according to the WHO designated regions

^c^ Each region was counted once for the single global SimEx report where all 6 WHO regions participated, therefore, the No. adds up to 61 instead of 56

^d^ Two SimEx reports had two types being used (e.g., a table top exercise followed by a drill), therefore the No. adds up to 58 instead of 56

^e^ AAR formats were not mentioned in 3 AAR reports

^f^ Each AAR may have one or more public health events reviewed (e.g., the country may have reviewed two epidemics at the same time). When multiple public health events were reviewed in a single AAR, events from the same public health event category was counted only once.

Among the 56 SimEx where reports were available, which included one report where all six WHO regions participated (each region was counted once each for these reports), 32 (52.5%) were from the AFR, followed by the EUR (*n* = 12, 19.7%), EMR (*n* = 8, 13.1%), WPR (*n* = 6, 9.8%), SEAR (*n* = 2, 3.3%), and AMR (n = 1, 1.6%; Table 2). Of the 56 SimEx reports received, 16 (28.6%) were conducted in 2016, 11 (19.6%) were conducted in 2017, 17 (30.4%) were conducted in 2018, and 12 (21.4%) were conducted in 2019 (Table 2). Among these 56 SimEx reports, the most common type of SimEx reported being used were table-top exercises (*n* = 36, 62.1%), followed by functional exercise (*n* = 10, 17.2%), field/full-scale exercises (n = 10, 17.2%), and drills (n = 2, 3.4%; Table 2). In two SimEx reports, two types of SimEx were used and were therefore counted individually.

**Fig. 1:**
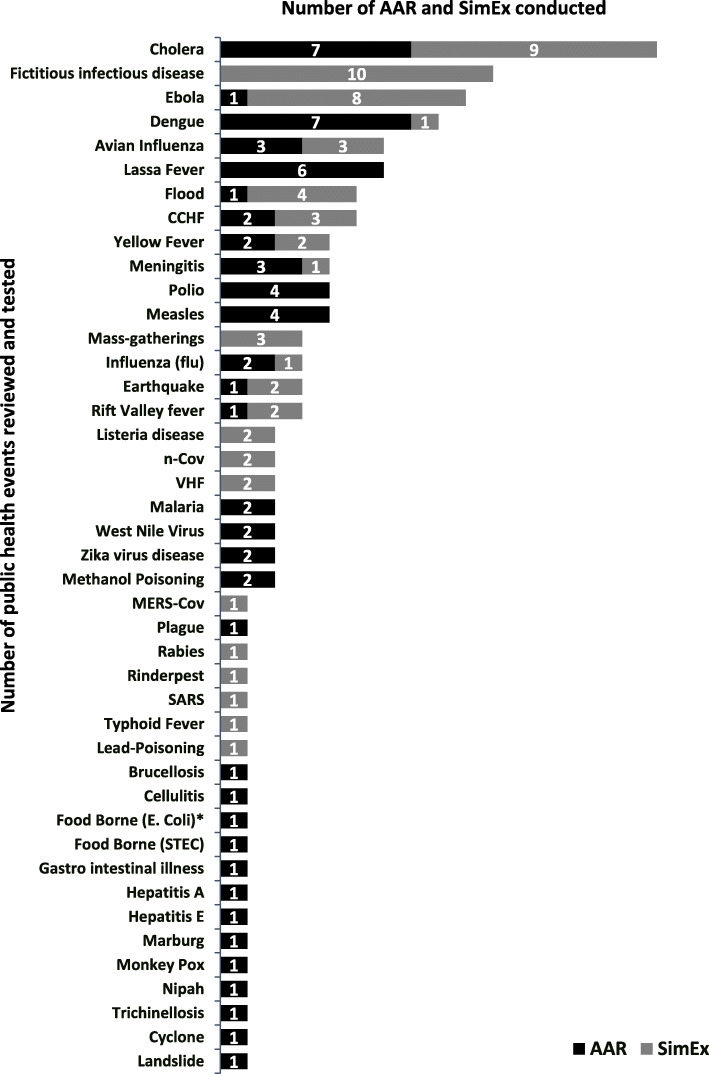
Public health events reviewed/tested in the WHO-supported AAR and SimEx conducted worldwide where reports were available, February 2016 to December 2019. *AAR, after action review; SimEx, simulation exercise; WHO, World Health Organization; CCHF, Crimean-Congo haemorrhagic fever; n-CoV, novel Coronavirus; VHF, Viral haemorrhagic fever; MERS-CoV, Middle East Respiratory Syndrome Coronavirus; SARS, Severe Acute Respiratory Syndrome; E. coli, Escherichia coli; STEC, Shiga toxin-producing Escherichia coli*

Of the 42 AAR and 56 SimEx, the most common PHE reviewed or scenario used respectively, were categorized as epidemics and pandemics (AAR: *n* = 38, 90.5%; SimEx: *n* = 46, 82.1%), followed by natural disasters (AAR: n = 3, 7.1%; SimEx: *n* = 6, 10.7%), and human-induced/societal related (AAR: n = 1, 2.4%; SimEx: n = 4, 7.1%; Table 2). When AAR and SimEx were examined individually, among the 42 AAR, the most common PHE reviewed were Cholera (*n* = 7, 16.7%), Avian influenza (n = 7, 16.7%), Lassa fever (n = 6, 14.3%), Poliomyelitis (n = 4, 9.5%), and Measles (n = 4, 9.5%; Fig. 1). In contrast, among the 56 SimEx, the most common PHE used as scenario were a fictitious infectious disease (*n* = 10, 17.9%), Cholera (*n* = 9, 16.1%), Ebola (*n* = 8, 14.3%), flood (n = 4, 7.1%), Avian influenza (*n* = 3, 5.4%), and Crimean-Congo hemorrhagic fever (CCHF) (*n* = 3, 5.4%; Fig. 1). With the AAR and SimEx examined collectively, the top six most common PHE reviewed or scenario used, were all epidemics and pandemics. The most common PHE used was Cholera (AAR: n = 7, 16.7%; SimEx: n = 9, 16.1%), followed by a fictitious infectious (AAR: N/A; SimEx: n = 10, 17.9%), Ebola (AAR: n = 1, 2.4%; SimEx: n = 8, 14.3%), Dengue (AAR: n = 7, 16.7%; SimEx: n = 1, 1.8%), Avian influenza (AAR: n = 3, 7.1%; SimEx: n = 3, 5.4%), and Lassa Fever (AAR: *n* = 6, 14.3%; SimEx: N/A; Fig. 1).

### IHR core capacity reviewed and tested in AAR and SimEx

After examining the 13 IHR core capacities reviewed and tested in AAR and SimEx, respectively, we found that 10 (76.9%) of the 13 IHR core capacities were reviewed at least once in AAR, with no AAR conducted among the reports received for food safety, chemical events, and radiation emergencies; all 13 (100%) IHR capacities were tested at least once in SimEx. For AARs, the number of IHR core capacities reviewed in AAR ranged between 3 and 8, with an average of 5.8 (SD = 1.35; data not shown). The number of IHR capacities validated in a SimEx ranged between 1 and 9, with an average of 4.6 (SD = 2.1; data not shown).

**Fig. 2:**
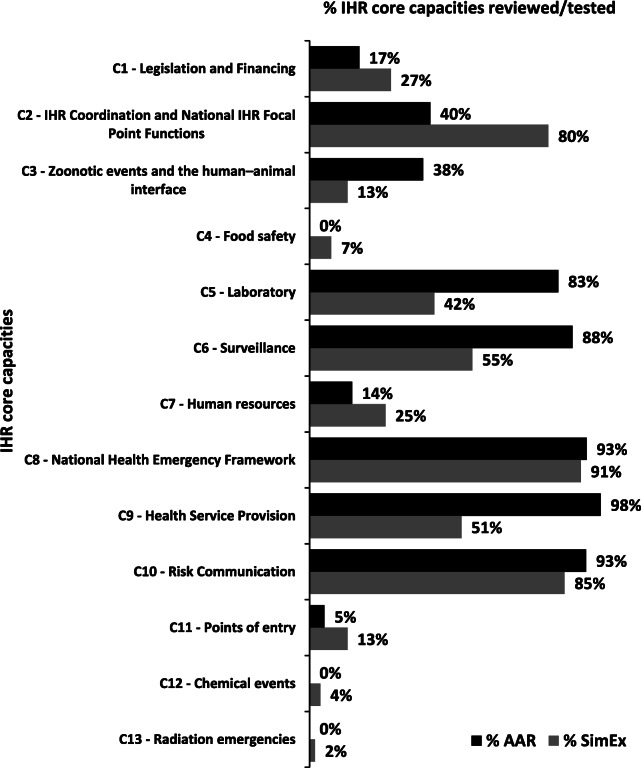
IHR core capacities reviewed/tested in the WHO-supported AAR and SimEx conducted worldwide where reports were available, February 2016 to December 2019. *AAR, after action review; SimEx, simulation exercise; WHO, World Health Organization*

For AAR, the most commonly reviewed IHR core capacities were health services provision (*n* = 41, 97.6%), risk communication (*n* = 39, 92.9%), national health emergency framework (*n* = 39, 92.9%), surveillance (*n* = 37, 88.1%) and laboratory (*n* = 35, 83.3%; Fig. 2). For SimEx, the most commonly tested IHR core capacity were national health emergency framework (*n* = 56, 91.1%), followed by risk communication (*n* = 48, 85.7%), IHR coordination and national IHR focal point functions (*n* = 45, 80.4%), surveillance (*n* = 31, 55.4%), health service provision (*n* = 29, 51.8%) and laboratory (*n* = 24, 42.9%; Fig. 2). The least commonly reviewed and tested IHR core capacities are radiation emergencies (AAR: N/A; SimEx: *n* = 1, 1.8%), chemical events (AAR: N/A; SimEx: n = 2, 3.6%), and food safety (AAR: N/A; SimEx: n = 4, 7.1%; Fig. 2); these three core capacities were not reviewed among the 42 AAR reports available.

### Strengths, challenges and recommendations identified during AAR and SimEx

We found that the overall strengths, challenges and recommendations documented in the AAR and SimEx reports, are aligned with the main IHR core capacities reviewed or tested, especially C8 - national health emergency framework, C9 – health service provision, and C10 - risk communication. The key strengths and challenges for these three IHR core capacities are shown in Table 3. The greatest strengths in national health emergency framework for both AAR and SimEx, were regular meetings and information sharing, good coordination between partners, and having coordination mechanisms in place. For health service provision, the greatest strengths were immediate notification following early detection and confirmation of a case, implementing large-scale vaccination for vulnerable people, and implementing safe and dignified burial. For risk communication, the greatest strengths were intersectoral communication, having existing communication structure in place, and information sharing with media and public. The main challenge reported under national health emergency framework for both AAR and SimEx included the lack of involvement of stakeholders, the lack of a unified command system, and insufficient coordination structure in place. For health service provision, the main challenges reported were inadequate infection prevention and control (IPC) practices and supplies, and inadequate case management and isolation of confirmed cases. For risk communications, the main challenge reported were poor community engagement, challenges with information sharing, and limited support from partners. When analyzing the recommendations of the three more commonly reviewed and tested IHR core capacities in AAR and SimEx collectively, we found that these recommendations generally aligned with the challenges identified in the reports. Some of the most frequently seen recommendations for national health emergency framework included to conduct regular trainings and exercises, and to develop an action plan and SOPs and define clear roles and responsibilities (data not shown). For health service provision, the most commonly seen recommendations included to ensure or improve isolation procedures and facilities, and to ensure implementation of IPC measures in healthcare facilities to prevent nosocomial infections (data not shown). Finally, for risk communication, the most commonly seen recommendations included to develop policies, plans and guides and to train health communicators, spokesperson and media (data not shown).

**Table 3:** Main strengths and challenges by key IHR core capacity reviewed/tested in the WHO-supported AAR and SimEx conducted worldwide where reports were available, February 2016 to December 2019

IHR Core Capacity	Strengths	Challenges
**C8 – National Health Emergency Framework**	1. Regular meetings and information sharing. 2. Good coordination between partners. 3. Coordination mechanism and structure in place.	1. Lack of stakeholders being involved and well briefed. 2. Lack of a unified command system and decision-making. 3. Insufficient coordination structure and systems in place.
**C9 - Health service provision**	1. Immediate notification, early detection and confirmation of case. 2. Large-scale vaccination for vulnerable people. 3. Safe and dignified burial implemented.	1. Inadequate IPC practices and supplies. 2. Insufficient care for cases. 3. Insufficient isolation of cases.
**C10 - Risk Communication**	1. Intersectoral communication with relevant stakeholders involved. 2. Existing communication structure or system in place. 3. Information sharing with media and public.	1. Poor community engagement. 2. Inefficient or inadequate information sharing. 3. Limited support from partners.

*AAR* after action review, *SimEx* simulation exercise, *WHO* World Health Organization

### Timeliness of WHO-supported AAR

**Fig. 3:**
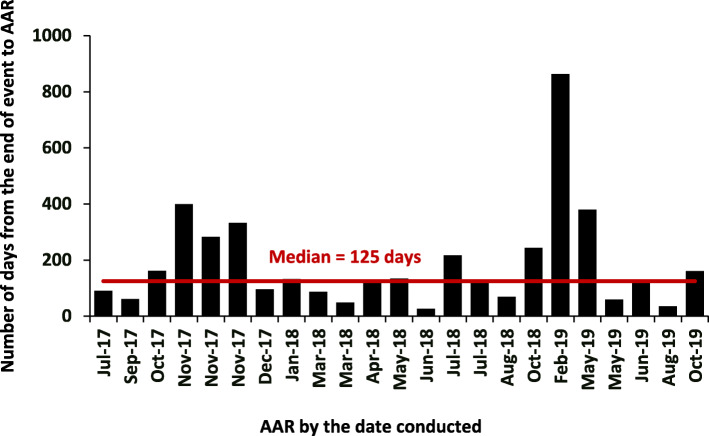
The timeframe from the end of a public health event to WHO-supported AAR conducted worldwide where reports were available, February 2016 to December 2019. *AAR, after action review; SimEx, simulation exercise; WHO, World Health Organization.* Note: Timeframe from the end of the declaration of the event to the AAR can only be calculated for 23 (55%) of the 42 AAR reports where dates of the end of the event were available

Given WHO recommends an AAR to be conducted as soon as possible and if possible within 3 months of the end of the event and/or of the response [5], we examined the time from the end of event/response to the AAR. Among the 42 AARs reports available, half (*n* = 23; 55%; data not shown) mentioned the date of the end of event or response. Among these 23 AAR conducted, the median timeframe between the end of the event and the AAR was 125 days (range = 25–399 days; Fig. 3).

## Discussion

The primary purpose of any AAR or SimEx is to identify and capture strengths and challenges in a structured manner and to propose concrete recommendations to improve plans, procedures and systems for emergency preparedness and response [5, 7, 8]. From 2016 to 2019, WHO supported AAR and SimEx in the six WHO regions that covered emerging and re-emerging infectious disease outbreaks, environmental and natural disasters, and societal crises [33]. Analyzing the extracted data from available AAR and SimEx reports produced some notable and clear trends. In this study, we showed AFR being the most common geographic region reporting, the vast majority of AAR format used being the working group format, and the most common type of SimEx used being the table top exercise. This study indicated that the predominant type of PHE reviewed or scenario used in AAR and SimEx were epidemics, with one SimEx testing a pandemic scenario involving all six regions, similar to the current COVID-19 pandemic. We also saw that in general, AAR were conducted later than the timeframe (from as soon as possible to 3 months from the end of the event) recommended by WHO to minimize recall bias [5]. In addition, in this study, we were able to link the events and functions reviewed/tested in AARs and SimEx to the 13 IHR core capacities, which can provide complementary information to other components in the IHR MEF and capacity building efforts. This, in turn, can offer a more comprehensive picture of the state of the public health preparedness and response in a given country, region and globally.

### The majority of the AAR/SimEx were conducted in the Africa region

In this study, we showed that more than half of the AAR and SimEx activities were conducted in AFR while other regions had far fewer reports available or even none. This may be due to a multitude of reasons. Firstly, the recent West Africa EVD outbreak in 2014–2016 and the EVD outbreak in North Kivu in the Democratic Republic of Congo, which were both declared as PHEIC [34, 35] have resulted in an enhanced priority for emergency preparedness and response in this region. In 2019, WHO AFR office (AFRO) supported four SimEx for EVD preparedness [36]. Secondly, given the WHO AFR encompasses many low-resource settings coupled with more infectious disease outbreaks and health emergencies, there has been a heavy emphasis by both WHO AFRO and countries in this region to strengthen their emergency preparedness and response capacity in light of the risks and vulnerabilities that are well recognized. Given the high frequency of acute public health event reported from AFR [37], there are more funding from the international community to conduct AAR and SimEx, with WHO AFRO more frequently been requested to support AAR and SimEx by their Member States compared to other WHO regions. Finally, other WHO regions such as AMR may have more experience and capacity to conduct these types of activities on their own without WHO support, and may not necessarily report their activities to WHO. In this study, we analyzed reports that were sent to WHO from WHO-supported AAR and SimEx. Given the increasing uptake of AAR and SimEx activities in the public health domain in recent years, we acknowledge that other SimEx or AAR were conducted by Member States that had the capacity and resources to undertake them on their own. In addition, it is also likely that some SimEx or AAR were done without WHO support but with support from other partner organizations, or simply not reported to WHO. Therefore, it is important to note that the data we presented may not represent all the AARs and SimEx conducted globally during the study period.

### Infectious disease outbreak as the predominant PHE reviewed and tested

This study identified that the majority of PHE that were reviewed in an AAR or used as a scenario in a SimEx were infectious disease outbreaks. WHO provides guidance and support to countries to strengthen all-hazards approaches to emergency preparedness which requires AAR and SimEx to be implemented for a range of threats beyond only infectious disease outbreaks, including health consequences from conflict, natural disaster, chemical or radio-nuclear spill and food contamination [1, 38]. As AAR and SimEx under the IHR MEF are mainly targeting the response capacity and capability of the Ministry of Health (MoH), it is reasonable to expect that this category is the most common PHE reviewed and tested. Although this may seem timely given the current COVID-19 pandemic, it is important to recognize that public health consequences are broad and do not exclusively come from infectious disease outbreaks alone. Countries are facing an increasing number of emergencies with health consequences from a broad range of hazards, including natural and human-made. In addition, many emergencies are complex, and can have significant public health, social, economic and political impacts. In practice, AAR and SimEx usually include health and non-health sectors as well as other partners and stakeholders. It is also important to emphasize that emergency management is not an exclusive responsibility of one sector or ministry alone.

### The difference in the focus of SimEx and AAR

In our study, the category “human-induced/societal” such as nuclear, chemical and mass gathering events was more likely to be used in SimEx, as compared to AAR. Our explanation for this difference can be found in the fact that these types of events are less common in real-world situations. As a general rule, each AAR and SimEx is tailored to be applicable to the national context or setting it is being implemented in as per their specific purpose and objectives. For AAR, the PHE review depends on the actual situation and what events are occurring in the country. In contrast, for a SimEx any event can be used as the scenario and the selection process is often guided by a multi-sectoral risk assessment to help identify the risks a country is most likely to face. Furthermore, these types of events may also be more sensitive from a security point of view, therefore, countries may wish to keep the reports classified or internal. It is therefore likely that there are even more exercises conducted for such events than reported to WHO.

We saw that in our study, the majority of the SimEx conducted were table top exercises. We hypothesize it is due to table top exercise being a discussion-based exercise that requires the least amount of resources and is the least complex to plan, implement and evaluate. Operational-based exercises such as drills, functional exercises, and field/full-scale exercises require more resources, including time needed, financial costs involved, and organizational experience necessary [8]. These operational exercises may be complex to plan and implement, and often require external support, especially in low-resource settings. WHO and partners have been promoting countries to increase the use of SimEx and to incorporate SimEx as a part of a comprehensive programme made up of progressively complex exercises, with each exercise building on the previous one. This `building block approach` is particularly important for organizations with less experience in conducting these activities, where it should start with basic table top exercises first, followed by progressively complex exercises requiring additional time and resources. Although our findings show the majority of SimEx being table top, it is unclear whether this is due to the fact that these are the least complex and require the least amount of resources, or because countries are adopting WHO’s advice of implementing comprehensive exercise programme’s building block approach. However, as part of a comprehensive exercise programme we hope to see a more equal distribution between discussion- based and more complex operational-based exercises being conducted in the coming years as countries familiarize themselves with SimEx.

### Cross-cutting IHR core capacities are more often reviewed

As seen in our study, since the scope, purpose and objectives of an AAR and SimEx can vary substantially, the number of IHR core capacities reviewed or tested can also differ significantly anywhere from one to multiple IHR core capacities reviewed or tested at the same time. Although it is up to the countries how many IHR core capacities they would like to review or test, for an AAR or SimEx to be most effective and useful, it is usually better to simplify and limit the scope by having only a few concrete objectives and a limited number of IHR core capacities to be reviewed or tested. This can help the AAR or SimEx to be more focused, resulting in more concrete outcomes that are more likely to be achieved.

For both AAR and SimEx, the most commonly reviewed IHR core capacities were similar, namely risk communication, IHR coordination, health service provision and national health emergency framework, such as the existence of a Public Health Emergency Operation Centre (PHEOC) or emergency preparedness and response plans. Consequently, the most common strengths and challenges were also linked to these main IHR core capacities reviewed or tested as anticipated. We hypothesize these IHR core capacities are most often reviewed or tested given their broad and cross-cutting nature, which often form the key elements in any emergency regardless of the type of PHE [39–41].

### Moving forward with proposed recommendations from AAR and SimEx

The proposed recommendations in AAR and SimEx were aligned with identified gaps in countries. However, the specificity of the priority recommendations vary per IHR core capacity, ranging from recommendations such as establishing or strengthening coordination mechanisms, ensuring or improving isolation for cases, to broader recommendations that can apply to different IHR core capacities, but if not concrete may not be as actionable, such as training and plan development. A major longstanding challenge after AAR and SimEx is accountability and the implementation of proposed recommendations. Although the involved stakeholders hold great expertise in identifying gaps, implementing measures on the local level, and genuinely learning lessons by implementing the needed steps, remains a persistent challenge. Therefore, post-AAR and post-SimEx follow up is vital to ensure expected or assumed improvements were made based on proposed recommendations. We believe the ideal way for recommendations to be implemented is to incorporate them into existing national plans such as the national action plan for health security (NAPHS) [42], and integrate them into national operational planning and budget cycles. WHO has also been developing additional guidance to better ensure that Simex and AAR include actionable lessons learned with identified lead implementers (including WHO and partner organizations), so that the recommended actions can be successfully implemented. This document titled, *“Roadmap for the implementation of recommendations from conducted IAR/AAR and SimEx*” is expected to be published on WHO website in quarter four of 2020 (*WHO, unpublished*).

### Benefits of AAR and SimEx

AAR and SimEx are recognized as key system improvement and learning tools in emergency management that help countries to assess and enhance their operational capability for public health preparedness and response [7]. Used by many organizations and across sectors, AARs and SimEx not only provide functional assessments but also play a key role in identifying strengths and gaps in the implementation of IHR core capacities. They can be used to review, validate or “stress test” the IHR core capacities reviewed by other IHR MEF instruments [4], for example, by looking at how effective a policy, plan or guideline is implemented, versus the existence of relevant policies, plans and guidelines. In this regard, AARs and SimEx are complementary to the other two IHR MEF components: SPAR [41] and JEE [43] as they provide a different perspective on how the IHR core capacities or response system functions in a “real” or simulated event.

Another critical benefit of AAR and SimEx is through the process of planning and conducting these activities, they can also build awareness of roles and responsibilities in different sectors involved in the PHE. These activities serve more than just identifying gaps and lessons learned. It can also start a cross-cutting dialogue across sectors and between individuals needed to strengthen preparedness and response to PHE. The AAR is an important learning tool and effective method for informing stakeholders of best practices, challenges and the root causes of preparedness gaps, and is used by many organizations and across sectors [6, 15, 16, 44–47]. Similar to the AAR, a high-fidelity simulation enables multiple learning objectives to be achieved in a realistic and secure context [48]. The use of simulation exercises involving the health community has also shown clear benefits on the individual level as well as on the organizational level, and are valuable and effective in the immediate, and to a lesser extend to the longer term [49]. The recommendations formulated during AAR and SimEx shed light for stakeholders and illuminate the way forward.

### Standardization of data & common principles for successful AAR & SimEx

The submitted reports were often not standardized, and there were inconsistencies in the structure, format, methodology and availability of key information. These discrepancies made it difficult to code and analyze findings, with some variables unable to be analyzed given only a minority of countries reported the information.

Nevertheless, various common principles were found to be essential for the successful planning, implementation and evaluation of AARs and SimEx. This includes clearly defined purpose, scope and objectives, having the right participants/organizations participate and having a structured evaluation and reporting process that ensures (national) ownership of the findings.

The purpose, scope and objectives are the foundation of any AAR and SimEx and should be carefully chosen to ensure the success of the activity in line with national priorities. Identifying and selecting the right participants is another crucial element for an AAR or SimEx and should be based on the purpose, scope and objectives, and thus on the functional areas or pillars that are reviewed or tested, respectively. If specific participants or agencies do not participate, inaccurate assumptions about the response functionality are likely to be made. WHO recommends countries to use a whole-of-society approach to ensure a broad commitment and mutual accountability in the support of and follow-up in implementations of recommendations emerging from AAR and SimEx. Furthermore, AAR and SimEx objectives should be linked with the capacities to be reviewed or tested, to ensure a well-structured evaluation process and report. Improvements in the design and implementation of SimEx and AARs could facilitate better reporting and measurement of preparedness outcomes [50, 51].

In line with above, WHO published the Country Implementation Guidance for After Action Reviews and Simulation Exercises under the IHR MEF [7]. This provides strategic guidance and criteria for inclusion of AAR and SimEx under the IHR MEF and introduces a structured evaluation method as well as a standardized minimum reporting template with timeline indicators for AARs. Furthermore, the Guidance for AAR and SimEx has also been published, which offers additional detail into the planning, execution and follow-up as of both activities [5, 8]. Using simple, standardized reporting format such as those provided by WHO and other partners [7, 14] will help consistent and standardized information collection for data analysis, which can, in turn, facilitate a comprehensive understanding about the Member States’ emergency preparedness and response capabilities under the IHR (2005).

### Limitations of study

There were several limitations to this study. Firstly, this study was limited by the fact that only a limited number of reports were available due to voluntary nature of AAR and SimEx under IHR MEF and therefore may not necessarily reflect a representative overview of all public health AAR and SimEx conducted globally. It is highly possible some countries with higher capacity conducted their own AAR and SimEx without informing WHO, therefore, were not included in this study. Secondly, as the study only used information provided in reports of activities. We did not have further secondary data to corroborate our findings, and that could be used as evidence to examine the impacts and benefits of AAR and SimEx. Thirdly, as WHO Member States may not have reviewed and tested the public health response pillar with explicit reference to the IHR core capacities, at times, it was challenging to code the response pillar reviewed and tested to the 13 IHR core capacities. Coding inconsistencies were addressed by having two independent coders and obtaining consensus with a team of WHO experts in public health emergency preparedness and response. Fourthly, overall, the timeframe from the end of an event to the AAR was longer than the 3 months WHO recommends [5], which may have resulted in some level of recall bias. Finally, in this study, it was evident the complexity of analyzing real-world public health practice data. In practice, one SimEx can involve one country, several countries in one region, or multiple countries in all WHO regions. For AAR, sometimes countries may request to conduct AAR for multiple public health events during the same AAR. These considerations may have been practical and useful for the countries and regions, but made the analysis more challenging when describing the data.

### Conclusions and recommendations

Every country faces a broad range of emergencies resulting from a variety of hazards that differ in scale, complexity and international consequences. In developed and developing countries alike, these emergencies can have extensive political, economic, social and public health impacts, with potential long-term consequences sometimes persisting for years after the emergency. AAR and SimEx are useful tools that can review PHE experienced by the country or simulate a rare PHE to facilitate individual and collective learning on the coordination and response of a future PHE should it arise.

From the analysis, it is fair to conclude that the strengths, challenges and recommendations all aligned with the functional areas or IHR capacities tested or reviewed. However, it is not possible to conclude whether these areas or IHR core capacities are indeed the key priority to invest in for enhancing public health preparedness and response. Future analysis may be useful, including the cross-analysis with other IHR MEF assessments available such as the SPAR and JEE results, and using proxy outcome measures such as reduction of morbidity and mortality, and timeliness of outbreak metrics to benchmark achievements.

Moving forward, it is vital to reiterate the importance of 1) scaling-up the implementation of SimEx and AAR as a means of enhancing preparedness; 2) reducing the timeframe from the end of an event and its AAR; 3) standardizing the critical information to be captured in AAR and SimEx and improving the ease and importance of information sharing with WHO; 4) clearly linking the public health response pillars tested and reviewed to the 13 IHR core capacities; 5) better defining the purpose, scope and objectives of the activity so results and the impact can be better measured; 6) Besides reviewing or testing health capacities in infectious disease outbreaks, encourage countries to review and test other hazards by adopting and promoting the all-hazard and multi-sectorial approach. With the recent guidance published by WHO [5, 7, 8], as well as from partner organizations [9–18]. we hope to see standardization in the AAR and SimEx methodology, practice and reporting.

### Current situation: AAR and SimEx in the context of the COVID-19 pandemic

Since December 2019, the COVID-19 pandemic has caused unprecedented global disruptions in all aspects of lives and livelihood of individuals, impacted global economic, trade and tourism, and pushed world leaders to rapidly come up with solutions to resolve this crisis [32]. We are at a critical juncture, as various public health measures are being implemented and their effects monitored. We are faced with a “new normal” in the way we conduct our daily lives for the months ahead until an effective and safe vaccine can be developed, and broadly and equitably distributed.

It is interesting to note that the main challenges we observed in our analysis of the AAR and SimEx reports from 2016 to 2019, such as poor community engagement, challenges with unified command and coordination system, and inadequate infection prevention and control (IPC) practices and supplies, including isolation of confirmed cases, were also some of the key challenges seen in the COVID-19 pandemic. As emphasized in the Global Preparedness Monitoring Board (GPMB) 2020 report [52], the GPMB calls for urgent actions for “*responsible leadership; engaged citizenship; strong and agile national and global systems for global health security; sustained investment in prevention and preparedness, commensurate with the scale of a pandemic threat; and robust global governance of preparedness for health emergencies*.”

As the number of COVID-19 cases and deaths decrease in certain countries, we urge affected countries to start preparing for IARs and AARs to ensure critical lessons can be learned, in preparation for future PHE. For the few countries, territories and areas which have none or very few COVID-19 cases and deaths, it could also be helpful to conduct COVID-19 SimEx to prepare for potential epidemics. In addition, COVID-19 SimEx can also be useful for those countries with more cases to scale up emergency operations and enhance preparedness capacities for possible next waves. Besides the existing generic guidance and tools for AAR and SimEx, WHO has published specific guidance on country COVID-19 IAR [26] as well as four COVID-19 specific SimEx packages. The COVID-19 IAR guidance and its ten accompanying tools were developed to support countries to conduct periodic review(s) of their national and subnational COVID-19 response efforts. The four COVID-19 SimEx packages include a generic SimEx that can be used at the national level, a health facility and IPC specific SimEx, a points of entry (PoE) specific SimEx, and a SimEx for the urban environments [53]. As of 2 October 2020, nine IARs have been conducted in AFR, SEAR and EUR and eight more are currently in the pipeline [54]. As of 14 October 2020, the WHO COVID-19 SimEx website has reached over 219,900 visitors since its first publication on 4 February 2020 (Copper, F.A. unpublished data). The GPMB has also highlighted in their 2020 report calls for urgent actions to countries to “*routinely conduct multisectoral simulation exercises to establish and maintain effective preparedness*” [52]. Both the COVID-19 IAR and SimEx packages have been added to the countries action checklist of the COVID-19 Partners Platform [55] to monitor and report which countries are conducting these activities and using them to update national COVID-19 preparedness and response plans.

The COVID-19 pandemic has affected both developed and developing countries irrespective of income level. This is a wake-up call to all countries that no country is immune to an emerging or re-emerging public health threat in our inter-dependent and inter-connected world. We urge all countries to invest in preparedness and incorporate the lessons from this pandemic to further advance national, regional and global health security.

## Supplementary information

**Additional file 1: Supplementary Table 1.** Variables extracted from WHO-supported and available After Action Review reports, February 2016 to December 2019. (DOCX 18 kb)

**Additional file 2: Supplementary Table 2.** Variables extracted from WHO-supported and available Simulation Exercise reports, February 2016 to December 2019.(DOCX 17 kb)

## Data Availability

Data may be available upon request to the corresponding author.

## Abbreviations

AAR: After action review
AFR: World Health Organization African region
AFRO: World Health Organization Regional Office for Africa
AMR: World Health Organization Region of the Americas
AMRO/PAHO: World Health Organization Regional Office for the Americas
CCHF: Crimean-Congo haemorrhagic fever
COVID-19: Coronavirus disease 2019
*E. coli*: *Escherichia coli*
EMR: World Health Organization Eastern Mediterranean Region
EMRO: World Health Organization Regional Office for the Eastern Mediterranean
EUR: World Health Organization European Region
EURO: World Health Organization Regional Office for Europe
EVD: Ebola virus disease
IHR: International Health Regulations (2005)
IHR MEF: International Health Regulations (2005) Monitoring and Evaluation Framework
IPC: Infection prevention and control
JEE: Joint External Evaluations
MERS-CoV: Middle East Respiratory Syndrome Coronavirus
MoH: Ministry of Health
NAPHS: National Action Plan for Health Security
n-CoV: Novel Coronavirus
PHE: Public health event
PHEIC: Public health emergency of international concern
PoE: Points of entry
SARS: Severe Acute Respiratory Syndrome
SBME: Simulation-based medical education
SD: Standard deviation
SEAR: World Health Organization South-East Asia Region
SEARO: World Health Organization South-East Asia Regional Office
SimEx: Simulation exercise
SOP: Standard operating procedure
SPAR: States Parties Annual Report
STEC: Shiga toxin-producing *Escherichia coli*
USD: United States Dollar
VHF: Viral haemorrhagic fever
WHA: World Health Assembly
WHO: World Health Organization
WPR: World Health Organization Western Pacific Region
WPRO: World Health Organization Regional Office for the Western Pacific
