# Supplementary material for: Simulation exercises and after action reviews – analysis of outputs during 2016–2019 to strengthen global health emergency preparedness and response
Source: Global Health. 2020 Dec 1;16:115. doi: 10.1186/s12992-020-00632-w (PMC7705853; doi:10.1186/s12992-020-00632-w)
Supplement: Supplementary file 2 — Additional file 2: Supplementary Table 2. Variables extracted from WHO-supported and available Simulation Exercise reports, February 2016 to December 2019.(DOCX 17 kb) [file 12992_2020_632_MOESM2_ESM.docx]

**Supplementary Table 2. Variables extracted from WHO-supported and available Simulation Exercise reports, February 2016 to December 2019.**

| **Variable** | **Variable type** | **Operational definition** |
| --- | --- | --- |
| **Participating Country** | String | Name of the country or countries that participated in the simulation exercise as a player. |
| **WHO region** | Categorical | Name of the WHO region or region(s) that had at least one of its country that participated in the simulation exercise as a player. |
| **Geographical scope** | Categorical | The geographical scope of the simulation exercise is classified from one of the four following options:   - Global: every WHO region had at least one country participating in the simulation exercise - Multi-countries: more than one country participated in the simulation exercise - National: only one country participated in the simulation exercise and entities or mechanisms established at the national level were tested during the exercise. - Sub-national: only one country participated in the simulation exercise and only entities or mechanisms established at the sub-national level were tested during the exercise. |
| **Year** | Numeric | Year during which the simulation exercise took place |
| **PHE used for scenario** | String | The name of a disease of the name of type of natural of man-made hazard at the origin of the Public Health event used for the simulation exercise scenario. |
| **PHE category** | Categorical | The public health event (PHE) categories are defined in WHO emergency response framework. There are three options possible based on the PHE used for scenario: Epidemics and Pandemics, Human induced / Societal; or Natural Disaster. |
| **Type of Simex** | Categorical | WHO simulation exercise manual defined four types of simulation exercise: tabletop exercise, drill, functional or full scale/field exercise. A specific exercise may combine different types |
| **C1 - Legislation and Financing** | Boolean | True is selected when at least one of the inject of the simulation exercise is testing the IHR core capacity “Legislation and Financing” as defined in State Party self-assessment annual reporting tool of the International Health Regulations (‎2005) |
| **C2 - IHR Coordination and National IHR Focal Point Functions** | Boolean | True is selected when at least one of the inject of the simulation exercise is testing the IHR core capacity “IHR Coordination and National IHR Focal Point Functions” as defined in State Party self-assessment annual reporting tool of the International Health Regulations (‎2005) |
| **C3 - Zoonotic events and the human–animal interface** | Boolean | True is selected when at least one of the inject of the simulation exercise is testing the IHR core capacity “Zoonotic events and the human–animal interface” as defined in State Party self-assessment annual reporting tool of the International Health Regulations (‎2005) |
| **C4 - Food safety** | Boolean | True is selected when at least one of the inject of the simulation exercise is testing the IHR core capacity “Food safety” as defined in State Party self-assessment annual reporting tool of the International Health Regulations (‎2005) |
| **C5 - Laboratory** | Boolean | True is selected when at least one of the inject of the simulation exercise is testing the IHR core capacity “Laboratory” as defined in State Party self-assessment annual reporting tool of the International Health Regulations (‎2005) |
| **C6 - Surveillance** | Boolean | True is selected when at least one of the inject of the simulation exercise is testing the IHR core capacity “Surveillance” as defined in State Party self-assessment annual reporting tool of the International Health Regulations (‎2005) |
| **C7 - Human resources** | Boolean | True is selected when at least one of the inject of the simulation exercise is testing the IHR core capacity “Human resources” as defined in State Party self-assessment annual reporting tool of the International Health Regulations (‎2005) |
| **C8 - National Health Emergency Framework** | Boolean | True is selected when at least one of the inject of the simulation exercise is testing the IHR core capacity “National Health Emergency Framework” as defined in State Party self-assessment annual reporting tool of the International Health Regulations (‎2005) |
| **C9 - Health Service Provision** | Boolean | True is selected when at least one of the inject of the simulation exercise is testing the IHR core capacity “Health Service Provision” as defined in State Party self-assessment annual reporting tool of the International Health Regulations (‎2005) |
| **C10 - Risk Communication** | Boolean | True is selected when at least one of the inject of the simulation exercise is testing the IHR core capacity “Risk Communication” as defined in State Party self-assessment annual reporting tool of the International Health Regulations (‎2005) |
| **C11 - Points of entry** | Boolean | True is selected when at least one of the inject of the simulation exercise is testing the IHR core capacity “Chemical events” as defined in State Party self-assessment annual reporting tool of the International Health Regulations (‎2005) |
| **C12 - Chemical events** | Boolean | True is selected when at least one of the inject of the simulation exercise is testing the IHR core capacity “Legislation and Financing” as defined in State Party self-assessment annual reporting tool of the  International Health Regulations (‎2005) |
| **C13 - Radiation emergencies** | Boolean | True is selected when at least one of the inject of the simulation exercise is testing the IHR core capacity “Radiation emergencies” as defined in State Party self-assessment annual reporting tool of the  International Health Regulations (‎2005) |
