# Supplementary material for: Simulation exercises and after action reviews – analysis of outputs during 2016–2019 to strengthen global health emergency preparedness and response
Source: Global Health. 2020 Dec 1;16:115. doi: 10.1186/s12992-020-00632-w (PMC7705853; doi:10.1186/s12992-020-00632-w)
Supplement: Supplementary file 1 — Additional file 1: Supplementary Table 1. Variables extracted from WHO-supported and available After Action Review reports, February 2016 to December 2019. (DOCX 18 kb) [file 12992_2020_632_MOESM1_ESM.docx]

**Supplementary Table 1. Variables extracted from WHO-supported and available After Action Review reports, February 2016 to December 2019.**

| **Variable** | **Variable type** | **Operational definition** |
| --- | --- | --- |
| Country | String | Name of the country for which the response is under review |
| WHO region | Categorical | Name of the WHO region in which the country is. |
| Geographical scope | Categorical | The geographical scope of the after action review is classified as one of the four following options:   - Global: every WHO region had at least one country involved in the response under review - Multi-countries: more than one country involved in the response under review - National: only country involved in the response and entities or mechanisms established at the national level were reviewed.   Sub-national: only one country covered by the review and only entities or mechanisms established at the sub-national level were reviewed. |
| Year | Numeric | Year during which the after action review took place |
| PHE under review | String | The name of a disease of the name of type of natural of man-made hazard at the origin of the Public Health event under review. |
| PHE category | Categorical | The public health event (PHE) categories are defined in WHO emergency response framework. There are three options possible based on the PHE under review: Epidemics and Pandemics, Human induced / Societal; or Natural Disaster. |
| Date End Outbreak | Date | When mentioned in the report, the date given for the official declaration by competent authority of the end of the outbreak |
| AAR format | Categorical | WHO guidance for After Action Review defined four format to conduct an after action review: debrief, key informants’ interviews, group work and mixed format. |
| Report language | String | Language used to write the report of the after action review |
| Report Status | Categorical | The status of the report is classified from one of the three following options:   - Final report published on Strategic Partnership for International Health Regulations (2005) and Health Security (SPH) - Final report made available to WHO but not public - Draft available to WHO but not public |
| Start date | Date | Day on which the after action review started |
| C1 - Legislation and Financing | Boolean | True is selected when the scope of the after action review included the IHR core capacity “Legislation and Financing” as defined in State Party self-assessment annual reporting tool of the International Health Regulations (‎2005) |
| C2 - IHR Coordination and National IHR Focal Point Functions | Boolean | True is selected when the scope of the after action review included the IHR core capacity “IHR Coordination and National IHR Focal Point Functions” as defined in State Party self-assessment annual reporting tool of the International Health Regulations (‎2005) |
| C3 - Zoonotic events and the human–animal interface | Boolean | True is selected when the scope of the after action review included the IHR core capacity “Zoonotic events and the human–animal interface” as defined in State Party self-assessment annual reporting tool of the International Health Regulations (‎2005) |
| C4 - Food safety | Boolean | True is selected when the scope of the after action review included the IHR core capacity “Food safety” as defined in State Party self-assessment annual reporting tool of the International Health Regulations (‎2005) |
| C5 - Laboratory | Boolean | True is selected when the scope of the after action review included the IHR core capacity “Laboratory” as defined in State Party self-assessment annual reporting tool of the International Health Regulations (‎2005) |
| C6 - Surveillance | Boolean | True is selected when the scope of the after action review included the IHR core capacity “Surveillance” as defined in State Party self-assessment annual reporting tool of the International Health Regulations (‎2005) |
| C7 - Human resources | Boolean | True is selected when the scope of the after action review included the IHR core capacity “Human resources” as defined in State Party self-assessment annual reporting tool of the International Health Regulations (‎2005) |
| C8 - National Health Emergency Framework | Boolean | True is selected when the scope of the after action review included the IHR core capacity “National Health Emergency Framework” as defined in State Party self-assessment annual reporting tool of the International Health Regulations (‎2005) |
| C9 - Health Service Provision | Boolean | True is selected when the scope of the after action review included the IHR core capacity “Health Service Provision” as defined in State Party self-assessment annual reporting tool of the International Health Regulations (‎2005) |
| C10 - Risk Communication | Boolean | True is selected when the scope of the after action review included the IHR core capacity “Risk Communication” as defined in State Party self-assessment annual reporting tool of the International Health Regulations (‎2005) |
| C11 - Points of entry | Boolean | True is selected when the scope of the after action review included the IHR core capacity “Chemical events” as defined in State Party self-assessment annual reporting tool of the International Health Regulations (‎2005) |
| C12 - Chemical events | Boolean | True is selected when the scope of the after action review included the IHR core capacity “Legislation and Financing” as defined in State Party self-assessment annual reporting tool of the  International Health Regulations (‎2005) |
| C13 - Radiation emergencies | Boolean | True is selected when the scope of the after action review included the IHR core capacity “Radiation emergencies” as defined in State Party self-assessment annual reporting tool of the  International Health Regulations (‎2005) |
